# Effect of Green Synthesized Iron Oxide Nanoparticles Using Spinach Extract on Triton X-100-Induced Atherosclerosis in Rats

**DOI:** 10.1155/2022/9311227

**Published:** 2022-10-07

**Authors:** Habila Obidah Abert, Hauwa Umaru Aduwamai, Saminu Shehu Adamu

**Affiliations:** ^1^Department of Biochemistry, Modibbo Adama University Yola, PMB 2076, Yola, Adamawa, Nigeria; ^2^School of Science, Adamawa State College of Education Hong, Hong, Nigeria; ^3^Department of Biochemistry, Ahmadu Bello University Zaria, Zaria, Nigeria

## Abstract

The effect of iron oxide nanoparticles (FeONPs) synthesized using *Spinacia oleracea* leaf extract on Triton X-100-induced atherosclerosis in white Wistar rats was determined. FeONPs were characterized to determine their size, structure, composition, and shape. *In vitro* antioxidant activity of FeONPs against 2, 2-diphenyl-1-picryl-hydrazyl-hydrate (DPPH) was determined. Atherosclerosis was induced by intraperitoneal administration of 5% Triton X-100 (100 mg/kg body weight) for 14 days. Group 1 received standard rat chow and water. Group 2 received 100 mg/kg body weight of Triton X-100 and a standard diet. Group 3 received 100 mg/kg body weight of Triton X-100 followed by 20 mg/kg body weight of atorvastatin for 21 days. Groups 4, 5, and 6 received 100 mg/kg body weight Triton X-100 was followed by variable concentrations of 100, 300, and 500 *µ*g/kg body weight FeONPs, respectively, for 21 days. Blood samples were analyzed for lipid, liver, antioxidant, and cardiovascular markers. Histopathology of the heart was also examined. Characterization revealed the amorphous nature, functional groups, and clustered topography of FeONPs. An upregulated antioxidant activity of FeONPs was observed in a dose-dependent manner. Administration of Triton X-100 showed elevated levels of lipid biomarkers except for high-density lipoprotein (HDL), which decreased in group 2 in comparison to group 1. Liver, antioxidant, and cardiovascular biomarkers all significantly increased. The structural alteration was observed in the heart tissue following histopathology examination. Administration of FeONPs significantly decreased all biomarkers and increased the level of HDL. Also, tissue architecture was restored. Our findings demonstrated that FeONPs were effective in ameliorating Triton X-100-induced atherosclerosis in rats.

## 1. Introduction

Cardiovascular diseases (CVDs) are the leading cause of death globally, claiming over 17 million lives annually and are projected to rise to 23.6 million by 2030 [1]. Although CVDs are multifactorial in nature, some important risk factors are considered the foremost causes. Among them, lifestyle-related factors (physical inactivity, poor nutrition and diet, obesity, and smoking) play the most significant roles in the prevalence of CVDs. Other health-related parameters such as high blood cholesterol levels, high blood pressure, diabetes mellitus, and metabolic syndrome, as well as family history and genetics, correlate with the occurrence of CVDs [2].

Atherosclerosis, a key pathogenesis of cardiovascular disease, is a disease characterized by the build-up of lipids', primarily cholesterol on the artery wall [3]. The advent of preventive medicine, secondary-prevention medication, and revascularization by interventional procedures such as balloon angioplasty and drug-eluting stents has led to considerable advances in the treatment of atherosclerosis. Despite the advances, atherosclerosis remains the major cause of morbidity and mortality in the field of cardiovascular disease globally [4].

Triton X-100 has been used to induce hyperlipidaemia (a major cause of atherosclerosis) in rats [5]. Triton acts as a surfactant and suppresses the action of lipases to block the uptake of lipoproteins from circulation by extrahepatic tissue, resulting in increased blood lipid concentration. Hyperlipidaemia is characterized by elevated serum total cholesterol, low-density cholesterol, very low-density lipoprotein cholesterol, and decreased high-density lipoprotein cholesterol levels [5].

Nanomedicine has been defined as the application of nanotechnology for the treatment, diagnosis, monitoring, and control of biological systems [6]. These applications include *in vivo* imaging and *in vitro* diagnostics, targeted delivery, biomaterials, and active implants. Despite its primary application being initially in cancer, nanomedicine has seen tremendous growth and extension beyond oncological applications in the twentieth century [7]. Although still in its infancy, nanomedicine is likely to have a substantial role in the management of cardiovascular diseases such as atherosclerosis.

When it comes to rapid development in the field of nanotechnology, nanoparticles (NPs) are at the forefront [8]. As the most common transition metal in the lithosphere (Earth crust), iron serves as the bedrock of modern infrastructure. However, when compared to same-group elements such as nickel, gold, cobalt, and platinum, iron oxides are somewhat neglected. Iron oxides are mostly used because they are inexpensive and play a critical role in several biological and geological processes. The green synthesis of iron oxide nanoparticles using plant materials has several benefits ranging from being inexpensive to its eco-friendliness and compatibility for several applications because it does not make use of toxic chemicals for the synthesis [9].

Green leafy vegetables such as spinach are rich sources of many nutrients and form a major vegetable group that has been designated as “nature's antiaging wonders,” having lots of medicinal value [10]. Spinach (*Spinacia oleracea*) belongs to the Chenopodiaceae family. It is one of the most important vegetables. It is a leafy cool-season vegetable with global cultivation, usually consumed after boiling or fresh as in salad [11, 12]. Phytochemicals and other bioactives present in spinach have the ability to scavenge reactive oxygen species and prevent macromolecular oxidative damage, modulate expression and activity of genes involved in metabolism, inflammation, proliferation, and defence against antioxidants. It curbs food intake by inducing the secretion of satiety hormones [13]. Spinach was used as a reducing agent for the green synthesis of iron oxide nanoparticle in this study and its effect (iron oxide nanoparticle) was determined on Triton X-100-induced atherosclerosis in white Wistar rats.

## 2. Materials and Methods

### 2.1. Drugs, Reagents, and Chemicals

Triton X-100, Atorvastatin (Gabbyto Pharmacy, Nigeria), liver function assay reagents (Randox kits), lipid profile assay reagents (Randox kits), total antioxidant status reagents (ELISA kits), reagents for cardiac markers (Dade Behring/Opus Plus), and histopathology reagents were used for the work. All reagents and chemicals were of analytical grade.

### 2.2. Plant Material and Experimental Animals

Fresh leaves of *Spinacia oleracea* (Spinach) were obtained from farms around the river Benue, Adamawa State, Nigeria. The plant's leaves were authenticated at the Plant Science Department of Modibbo Adama University, Yola.

Thirty male white Wister rats (weighing between 180 ± 20 g/body weight) were used for the study. The rats were procured from the National Veterinary Research Institute, Vom, Plateau State. The rats were kept in cages barred with steel nets and allowed to acclimatize for 14 days. They were constantly supplied with rat chow (Vital Feed, Jos) and clean water *Ad libitum*. The Research and Ethics Committee of the Department of Biochemistry, Modibbo Adama University, Yola, Adamawa State, Nigeria, approved the overall handling and management of the experimental animals.

### 2.3. Preparation of Plant Sample

The freshly collected leaves of *Spinacia Oleracea* were cleaned under running water and washed with deionized water. The leaves were carefully separated from the stem, air-dried at room temperature, and grounded into powder. For the extract production, 100 g of powdered *Spinacia oleracea* sample was boiled with double distilled water (1000 ml) in an Erlenmeyer flask while being continuously stirred for 15 minutes. The extract was cooled to room temperature and filtered using Whatman filter paper number 1 (Sigma Aldrich).

### 2.4. Green Synthesis of Iron Oxide Nanoparticles

The green synthesis of iron oxide nanoparticles using *Spinacia oleracea* leaf and iron chloride as a precursor was carried out according to the method described by Amutha and Sridhar [14]. FeCl_3_·6H_2_O and FeCl_2_·4H_2_O (1 : 2 molar ratios) were dissolved in 100 ml of distilled water in a 250 ml beaker and heated at 80°C on a hot plate with mild stirring using a magnetic stirrer. After 10 minutes, 20 ml of aqueous solution of the *Spinacia oleracea* extract was added to the mixture. A visible colour change (from light yellow to dark brown) was observed. After 10 minutes, 20 ml of aqueous solution of sodium hydroxide was added to the mixture at a rate of 3 ml per minute to allow for uniform precipitation of the iron oxide. The mixture was allowed to cool down to room temperature, and the iron oxide nanoparticles were obtained by decantation. The iron oxide formed was washed thrice with distilled water and dried at room temperature.

### 2.5. Characterization of Iron Oxide Nanoparticles Synthesized Using *Spinacia oleracea* Leaf

#### 2.5.1. Size and Structure

X-ray diffraction analysis (XRD) was used to determine the size and structure of the synthesized iron oxide nanoparticles according to the method described by Boukhoubza et al. [15]. The synthesized iron oxide nanoparticles were centrifuged at 10,000 rpm for 15 minutes, and the pellets were redispersed in sterile double distilled water and centrifuged at 10,000 rpm for 10 minutes. The purified pellets were dried at 50°C in an oven and analyzed by an X-ray Diffraction Unit (XRD) (Pan Analytical, X-pert pro, Netherland). The X-ray diffraction (XRD) measurement of iron oxide nanoparticles synthesized by leaves of *Spinacia oleracea* was carried out using a Cu-K*α* radiation source in the scattering range of 20-80 on the instrument operating at a voltage of 45 kV and a current of 40 mA. The grain size of synthesized iron oxide nanoparticles and their structure were determined by X-ray diffraction spectroscopy. The particle size of the prepared samples was determined by using Scherrer's equation.

#### 2.5.2. Shape

Scanning electron microscopy (SEM) was used to determine the shape of the iron oxide nanoparticles according to the method described by Forough and Farhadi [16]. The synthesized nanoparticles were dispersed in double distilled water, and the resultant suspension was homogenized using an ultrasonicator for two hours. A drop of the nanoparticle's suspension was placed on a piece of microglass slide attached to a metal grid coated with carbon film and dried at room temperature. The sample was sputter coated with gold and visualized with a JEOL JSM-6480 LV SEM to assess the shape.

#### 2.5.3. Composition

FTIR spectroscopy (IR Prestige21, Shimadzu, Pvt Ltd, Japan) measurement using the KBr pellet method (1/8 of the solid sample (iron oxide nanoparticles) and 0.50 teaspoons of KBr thoroughly mixed in a mortar by grinding using a pestle) was used to determine the chemical composition of the iron oxide nanoparticles as described by Kero et al. [17].

### 2.6. *In vitro* Determination of Iron Oxide Nanoparticle Scavenging Activity against DPPH

The determination was made according to the method described by Trinh et al. [18]. The sample extract (0.2 millilitre [ml] iron oxide nanoparticle solution) was diluted with methanol, and 2 ml of DPPH solution (0.5 mM) was added. After 30 min, the absorbance was measured at 517 nm. The percentage of the DPPH radical scavenging was calculated using the equation given below.

(1) $$\begin{array}{c} \text{\%DPPH RSA}=\frac{\text{Abs}{\text{.}}_{\text{control}}-\text{Abs}{\text{.}}_{\text{sample}}}{\text{Abs}{\text{.}}_{\text{control}}}\times 100, \end{array}$$

where Abs._control_ is the absorbance of control and Abs._sample_ is the absorbance of the sample. RSA is the radical scavenging activity.

### 2.7. Induction of Atherosclerosis

Atherosclerosis was induced in rats by intraperitoneally administering high doses of 5% Triton X-100 (100 mg/kg body weight) for 14 days [5].

### 2.8. Atherosclerosis Study

Prior to the main study, a pilot study involving 6 rats was carried out to determine the concentration that would be effective for treatment using FeONPs, having already established the concentration that is effective for induction of atherosclerosis in a previous study. Concentrations of 100, 200, and 300 *µ*g/kg bw FeONPs for 21 days were initially utilized as treatment. There was no significant difference between the concentrations of 100 and 200 *µ*g/kg bw FeONPs in terms of biochemical parameters that were examined. However, a significant difference was observed between the concentrations of 100 and 300 *µ*g/kg bw FeONPs, which informed our decision about the concentrations that were used as treatment. This implies that higher doses of FeONPS will even be more effective in atherosclerosis studies.

The experimental animals were grouped into 6 of 5 rats each. The rats were treated differently as shown in the table below.

  Group-1: Sham − Standard diet and water.
  Group-2: Negative Control − 100 mg/kg bw Triton X-100 for 14 days + standard diet for 21 days.
  Group-3: Positive Control − 100 mg/kg bw Triton X-100 for 14 days + standard diet + 20 mg/kg bw atorvastatin for 21 days.
  Group-4: Treatment 1 − 100 mg/kg bw Triton X-100 for 14 days + standard diet + 100 *µ*g/kg bw FeONPs for 21 days.
  Group-5: Treatment 2 − 100 mg/kg bw Triton X-100 for 14 days + standard diet + 300 *µ*g/kg bw FeONPs for 21 days.
  Group-6: Treatment 3 − 100 mg/kg bw Triton X-100 for 14 days + standard diet + 500 *µ*g/kg bw FeONPs for 21 days.

After the last dose, the rats were made to fast overnight. They were sacrificed using urethane as anaesthesia, and blood samples were collected by cardiac puncture into sample bottles. Serum was obtained from the blood with the aid of a benchtop centrifuge and was used for the estimation of biochemical parameters. ***Lipid Profile Parameters*** (total cholesterol [TC]—based on CHOD-PAP enzyme colorimetric method [19]; triglycerides [TG]—based on the GPO-PAP enzyme colorimetric method [20]; high-density lipoprotein [HDL]—direct measurement method [21]; low-density lipoprotein [LDL]—based on a two reagent system method as described by Martínez–Morillo et al. [22]; and Atherogenic Index [AI]—by calculation as described by Kammar-García et al. [23]), ***liver function markers*** (alanine amino transferase [ALT] and aspartate transaminase [AST]—standard method described by Frankel and Reitman [24];and alkaline phosphatase [ALP]—as described by Adeyemi et al. [25]), ***serum antioxidant markers*** (superoxide dismutase [SOD]—based on the generation of superoxide radicals produced by xanthine and xanthine oxidase, as described by Misra and Fridovich [26]; glutathione peroxidase [GPx]—as described by Flohe and Gunzler [27]; and catalase [CAT]—measured spectrophotometrically as described by Koroliuk et al. [28]), and ***serum cardiovascular markers*** (cardiac troponin I—based on fluorescence immunoassay as described by Boditech Med Incorporated [29]; myoglobin concentration-based on fluorescence immunoassay [30]; and creatinine phosphokinase isoenzyme MB CK-MB—also based on a fluorescence immunoassay [31]) were determined. The histopathology procedure was carried out as described by Ragavan and Krishnakumari [32]. Briefly, heart tissue was harvested and processed for photomicrographic examinations. The tissue was fixed on removal from the animal in 10% neutral buffered formalin. The tissue was kept in the fixative for 12 hours, dehydrated with serial ethanol cycles (absolute), and then embedded in paraffin. The paraffin-embedded tissue was cut into 5-*μ*m sections. The tissue section was deparaffinized and stained with Mayer hematoxylin and eosin stains for light photomicroscopic capture.

### 2.9. Statistical Analysis

A one-way analysis of variance (ANOVA) was used. The grouped data were expressed as mean ± standard error of the mean (SEM); the statistical significance of the difference was evaluated using Statistical Package for Social Sciences (SPSS) Version 24.

## 3. Result

### 3.1. Green Synthesis of Iron Oxide Nanoparticles

A rapid colour change from light yellow to black was observed after the addition of one-part *Spinacia oleracea* extract to 9 parts of iron (III) chloride hexahydrate. The metal ions were reduced after being exposed to an aqueous extract of *Spinacia oleracea* leaf extract within 24 hours. The change in colour from light yellow to black was the initial visual confirmation of the presence of iron oxide nanoparticles.

### 3.2. X-Ray Diffraction (XRD) Analysis of Iron Oxide Nanoparticles

**Figure 1:**
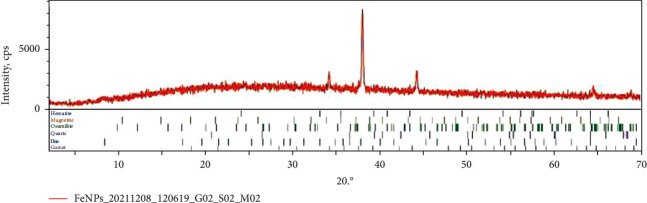
X-ray diffraction spectrum of iron oxide nanoparticles.

Figure 1 shows the XRD spectrum of synthesized iron oxide nanoparticles. The spectrum showed that the pattern is deficient in distinctive diffraction peaks. However, a broad peak, which is a characteristic diffraction line of iron oxide nanoparticles, is seen at around 2Ѳ of 38.4169^0^, indicating the nature of the synthesized iron oxide nanoparticles as amorphous.

### 3.3. Fourier Transform Infrared Spectroscopy (FTIR) Analysis of Aqueous Leaf Extract of *Spinacia oleracea*

**Figure 2:**
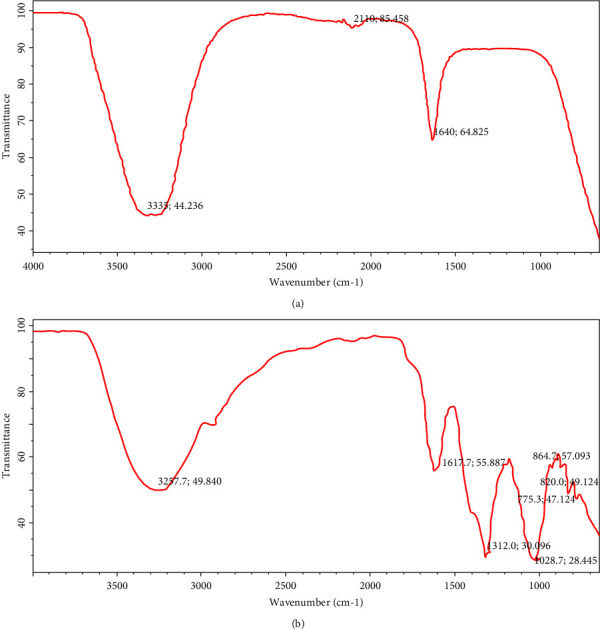
(a) FTIR spectrum of *Spinacea oleracea* leaf extract. (b) FTIR spectrum of synthesized iron oxide nanoparticles.

Figure 2(a) shows the FTIR spectrum of *Spinacia oleracea* leaf extract. The FTIR spectrum showed some prominent peaks at 3335 cm^−1^, which corresponds to the OH stretching vibration of the hydroxyl group present in polyphenols, 2110 cm^−1^ corresponding to the alkyne terminal, and 1640 cm^−1^ corresponding to the alkenyl stretching frequency, all of which are secondary metabolites of the extract.

### 3.4. Fourier Transform Infrared Spectroscopy (FTIR) Analysis of the Synthesized Iron Oxide Nanoparticles

Figure 2(b) shows the FTIR spectrum of synthesized iron oxide nanoparticles. The spectrum showed absorption peaks at 3257.7 cm^−1^ corresponding to O-H stretching frequency, 1617.7cm^−1^and 1312.0 cm^−1^ corresponding to carboxylate functional groups, 775.3 cm^−1^ corresponding to aliphatic chloro-compounds, 1028.7 cm^−1^ corresponding to primary amine, 820.0 cm^−1^ corresponding to C-O-O stretching frequency, and 864.7 cm^1^ which corresponds to aromatic stretch. A trend in the inclination of peak intensities of functional groups was observed on the spectrum, which could be attributed to the interaction of functional groups with the secondary metabolites of spinach extracts serving as stabilizing agents.

### 3.5. Scanning Electron Micrography Micrographs of the Synthesized Iron Oxide Nanoparticles

**Figure 3:**
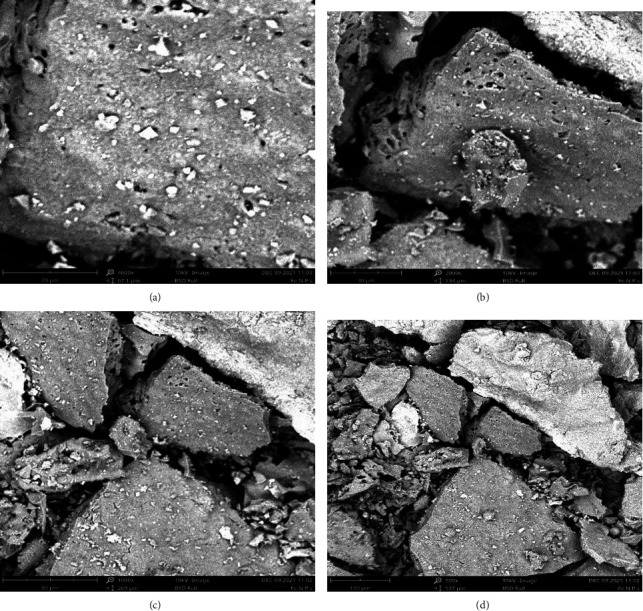
SEM images of synthesized Iron oxide nanoparticles at (a) 20* μ*m, (b) 30* μ*m, (c) 80* μ*m and (d) 100* μ*m respectively.

Figure 3 shows the SEM micrographs of synthesized iron oxide nanoparticles having clustered topography as well as variable surface morphology in terms of shape and size due to agglomeration, confirming their nature as amorphous.

### 3.6. DPPH Free Radical Scavenging Activity of Iron Oxide Nanoparticles

Table 1 shows the free radical scavenging activity of iron oxide nanoparticles against DPPH. The result showed that the antioxidant potential of iron oxide nanoparticles was upregulated. The upregulation was dose-dependent, as seen in the table. However, in comparison to ascorbic acid at the same concentration, the DPPH scavenging activity of iron oxide nanoparticles was lower.

### 3.7. Effect of Iron Oxide Nanoparticles on Lipid Profile Parameters

Table 2 shows the effect of iron oxide nanoparticles on lipid profile parameters. There was a significant increase (*p* < 0.05) in the levels of TC, TG, LDL, and AI, as well as a significant decrease in the level of HDL in the negative control group (rats administered with 100 mg/kg body weight of Triton X-100) compared to the sham group. Administration of iron oxide nanoparticles however ameliorated the hyperlipidaemic effect of Triton X-100 by lowering the levels of TC, TG, LDL, and AI and increasing HDL as observed in all iron oxide nanoparticle treated groups. The amelioration was dose-dependent as seen in the table in which the highest antihyperlipidemic activity was observed in the 500 *µ*g/kg body weight iron oxide nanoparticles-treated group. However, in contrast to the atorvastatin-treated group, the reduction in the blood concentration of lipids by iron oxide nanoparticles is significantly lower.

### 3.8. Effect of Iron Oxide Nanoparticles on Liver Biomarkers

Table 3 shows the effect of iron oxide nanoparticles on liver biomarkers. There was a significant increase (*p* < 0.05) in the activities of AST, ALT, and ALP in the negative control group compared to sham following the administration of Triton X-100. Administration of iron oxide nanoparticles however decreased their levels (AST, ALT, and ALP) across all treatment groups in a dose-dependent manner.

### 3.9. Effect of Iron Oxide Nanoparticles on Serum Antioxidant Markers

Table 4 shows the effect of iron oxide nanoparticles on serum antioxidant markers. The activities of SOD, CAT, and GPx were significantly decreased (*p* < 0.05) in Triton X-100-treated group compared to the sham group. Administration of iron oxide nanoparticles however significantly increased (*p* < 0.05) the activity of SOD and CAT but with no statistically significant increase in the activity of GPx across all treatment groups. The effects of iron oxide nanoparticles on SOD and CAT at a dose of 500 *µ*g/kg were found to be comparable with those of the standard drug atorvastatin.

### 3.10. Effect of Iron Oxide Nanoparticles on Cardiovascular Markers

Table 5 shows the effect of iron oxide nanoparticles on serum cardiovascular biomarkers. The activities of creatinine kinase, troponin I, and myoglobin were significantly increased (*p* < 0.05) in Triton X-100-treated group (negative control) compared to the sham group. Administration of iron oxide nanoparticles was marked by a decrease in the levels of creatinine kinase, troponin, and myoglobin across all treatment groups in comparison to the negative control group.

### 3.11. Effect of Iron Oxide Nanoparticles on Cardiac Histopathology

**Figure 4:**
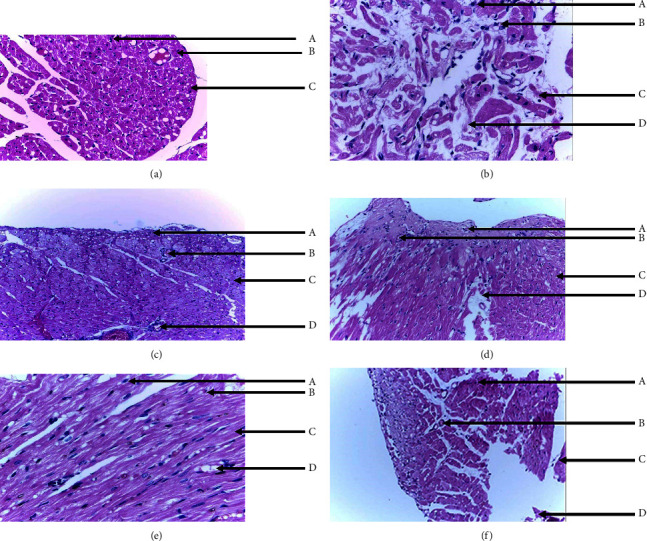
(a) Cross section of the heart of sham group. (b) Cross section of the heart of negative control group. (c) Cross section of the heart of standard control group. (d) Cross section of the heart of 100*μ*g/kg body weight FeONPs group. (e) Cross section of the heart of 300*μ*g/kg body weight FeONPs group. (f) Cross section of the heart of 500*μ*g/kg bw FeONPs group.

Figures 4(a)-4(f) show the effect of iron oxide nanoparticles on cardiac histopathology. The heart section of group 2 (negative control) showed marked fatty infiltration in cardiomyocytes, altered tissue architecture, and necrotic changes compared to group 1 (sham), which showed normal tissue architecture, no fatty changes, and normal morphology of myocardial cells. Administration of iron oxide nanoparticles showed very mild fatty infiltration and restored the cardiomyocytes as seen at the highest dose of 500 *µ*g/kg bw FeO NPs.

Figure 4(a) shows the cross section of the heart of the sham group stained with haematoxylin and eosin. Section (H and E, ×40) shows no fatty changes with normal tissue architecture, multiple peripheral nuclei, and regular morphology of myocardial cell membrane. KEY: A = cardiomyocytes, B = peripheral nuclei, C = myocardial cell membrane.  Figure 4(b) shows the cross section of the heart of the negative control group stained with haematoxylin and eosin. Section (H and E, ×40) shows alteration in tissue architecture and focal fatty infiltration in myocardial cells. KEY: A = cardiomyocytes, B = peripheral nuclei, C = myocardial cell membrane, D = fatty infiltrates.  Figure 4(c) Shows the cross section of the heart of standard control group stained with haematoxylin and eosin. Section (H and E, ×40) shows restored tissue and myocardial architectures. KEY: A = cardiomyocytes, B = peripheral nuclei, C = myocardial cell membrane, D = very mild fatty infiltrates.  Figure 4(d) shows the cross section of the heart of 100 *µ*g/kg bw group stained with haematoxylin and eosin. Section (H and E, ×40) shows focal fatty infiltration in myocardial cells. KEY: A = cardiomyocytes, B = peripheral nuclei, C = myocardial cell membrane, D = fatty infiltrates.  Figure 4(e) shows the cross section of the heart of the 300 *µ*g/kg bw group stained with haematoxylin and eosin. Section (H and E, ×40) shows a fair effort at regenerating myocardial cells. KEY: A = cardiomyocytes, B = peripheral nuclei, C = myocardial cell membrane, D = mild fatty.  Figure 4(f) shows the cross section of the heart of the 500 *µ*g/kg bw group stained with haematoxylin and eosin. Section (H and E, ×40) shows positive cardiomyocytes restoration with very mild fatty infiltration. KEY: A = cardiomyocytes, B = peripheral nuclei, C = myocardial cell membrane, D = very mild fatty infiltrates

## 4. Discussion

Iron oxide nanoparticles were successfully synthesized by a biological method using *Spinacia oleracea* (Spinach) leaf extract and ferric chloride solution at a ratio of 1 : 9. The addition of one-part spinach leaf extract to nine parts of 1 mM aqueous FeCl_3_ solution instantly turned dark brown from its original pale yellow. The metallic ions present in the ferric chloride solution were reduced to iron oxide after being exposed to aqueous leaf extract of *Spinacia oleracea.* The change in colour indicated the formation of iron oxide nanoparticles. Similar result on the formation of nanoparticles using castor leaf extract [33] and grape leaf [34] were reported. Likewise, various plant extracts such as *Moringa oleifera* [35], *Lagenaria siceraria* [36], *Mangifera indica, Murraya Koenigii, Azadiracta indica,* and *Magnolia champaca* [37] have been reported for the synthesis of iron oxide nanoparticles. Such rapidly processed plant-mediated iron metallic nanoparticles are an alternative to chemical synthesis protocols and can serve as a low-cost reductant for synthesizing iron nanoparticles.

Figure 1 shows the X-ray diffraction (XRD) pattern of the biologically synthesized iron oxide nanoparticles using the aqueous leaf extract of spinach. The XRD pattern showed a broad peak at 2*θ* = 34.4169^0^, whereas the rest of the patterns were insufficient in distinctive diffraction peaks. The result indicated that the formed iron oxide nanoparticles were predominantly amorphous in nature, which is also in agreement with reported literature [38]. The obtained insufficient distinctive XRD peaks could be indexed to a rhombohedral structure, which is in accordance with the file NO 89-8104 of the Joint Committee on Power Diffraction Standards (JCPDS). A broad peak presented at 2*θ* = 34.4169^0^ could be because of coated organic materials from the reaction media, which is responsible for stabilizing the synthesized nanoparticles.

Figure 2(a) shows the FTIR spectrum of the spinach leaf extract for the identification of biomolecules responsible for the formation of iron oxide nanoparticles. The spectrum showed bands at 3352 cm^−1^, 2110 cm^−1^, and 1640 cm^−1^. The intense broad absorption at 3352 cm^−1^ is designated to the -OH stretching vibration of the hydroxyl group, demonstrating the existence of hydrogen bonds or carboxylic acids present in polyphenols. Phenolic compounds such as phenolic acids, flavonoids, and tannins are reducing agents widely distributed in plants and have been the focus of great attention because of their antioxidant activities, which potentially have useful implications for the health of human beings [39]. The peak at 2110 cm^−1^ corresponds to the alkyne C≡C terminal, while the peak at 1640 cm^−1^ confirms the presence of alkenyl C=C stretching frequency. The differences in wavenumbers are mainly due to different hybridization and the number of ligands on the carbon atom.

Figure 2(b) shows the FTIR spectrum of the synthesized iron oxide nanoparticles. The spectrum showed absorption peaks at 3257.7 cm^−1^ corresponding to hydroxy compounds, 1617.7cm^−1^and 1312.0 cm^−1^ corresponding to carboxylate functional groups, 775.3 cm^−1^ corresponding to aliphatic chloro-compounds, 1028.7 cm^−1^ corresponding to primary amine, 820.0 cm^−1^ corresponding to C-O-O stretching frequency, and 864.7 cm^1^ which corresponds to aromatic stretch. However, a trend in inclination of peak intensities of functional groups was observed that could be attributed to the interaction of functional groups with the stabilizing agents of spinach extract. The results obtained are in complete agreement with the previously reported work [37], which found a trend in the inclination of peak intensities of functional groups and attributed it to the interaction of the functional groups with the stabilizing agents of different leaf extracts used in the synthesis of iron oxide nanoparticles. Due to their low surface charge, iron oxide nanoparticles (FeONPs) tend to accumulate in an aqueous medium. Consequently, citrate is used as a stabilizing agent in the chemical synthesis of nanoparticles [40]. Aqueous extract of plants coated/stabilized with FeONPs by low molecular weight organic acids (such as citrate, malate, and oxalate) prevents the interactions of FeONPs together and enhances the interactions with water molecules, resulting in more colloidal stability.

Figure 3 shows the scanning electron microscopy (SEM) micrograph of the synthesized iron oxide nanoparticles. The micrographs were recorded at magnifications of 20 *µ*m, 30 *µ*m, 80 *µ*m, and 100 *µ*m, as seen above. The surface morphology of the synthesized iron oxide was characterized using SEM. A topographical view of the micrograph showed that iron oxide nanoparticles were clustered and varied in shape and size due to agglomeration. The reason for the agglomeration of iron nanoparticles may be due to the magnetic nature, high surface-to-volume ratio of the nanoparticles, and the presence of biomolecules in *Spinacia oleracea* responsible for the capping of the iron oxide nanoparticles [41].

Table 1 shows the free radical scavenging activity of iron oxide nanoparticles against DPPH. Five different concentrations of both the sample (iron oxide nanoparticles) and standard (ascorbic acid) (20, 40, 60, 80, and 100 *µ*g/ml) were used. Although there was an upregulation in the antioxidant activity of both iron oxide nanoparticles and ascorbic acid, that of iron oxide nanoparticles was not as pronounced as ascorbic acid. The upregulation was done in a dose-dependent manner, as seen in the table. A similar work was reported by Sandhya and Kaleiselvam [42]. Since aqueous leaf extract of *Spinacia oleracea* is available in the synthesized iron oxide nanoparticles, it may be concluded that some of the phenolic compounds involved in the capping of iron oxide nanoparticles can quench the reactive oxygen species by acting as reducing/oxidizing agents [43]. The green synthesized iron oxide nanoparticles thus have the potential to be utilized in a host of biomedical applications because of their high cytocompatibility and antioxidant activity. The bio entity surface functionalization can be used to modify the properties of nanoparticles with regard to chemical functionality and applications [44]. The pharmacognostic properties of the phytochemicals enhance the therapeutic properties of the nanoparticles with improved biological activity [45].

Table 2 shows the effect of iron oxide nanoparticles on lipid profile parameters. The result of the study corroborated the findings of Parwin et al. [5] in which there was a significant increase (*p* < 0.05) in the levels of TC, TG, LDL, and AI, as well as a significant decrease (*p* < 0.05) in the level of HDL in the negative control group (rats administered with 100 mg/kg body weight of Triton X-100) compared to the sham group. Triton X-100 suppresses the action of lipoprotein lipase and blocks the uptake of lipoproteins from circulation by the extrahepatic tissues, resulting in increased blood lipid concentrations [46]. Elevated levels of total cholesterol are associated with an increased risk of atherosclerosis. High levels of triglycerides and LDL are associated with coronary artery disease [47]. Animal studies on the role of iron oxide nanoparticles in experimental atherosclerosis are limited. Although one study suggested that excessive iron loading in hypercholesterolemic rats was beneficial and significantly reduced lesion formation [47].

Polyphenols contribute to hypolipidaemic activity by increasing cholesterol metabolism and by modulating the enzymes involved in cholesterol metabolism, such as 3-hydroxy-3-methylglutaryl coenzyme A (HMG-CoA), lecithin cholesterolacyl transference, cholesterol 7*α*-hydroxylase, and acyl coA: cholesterol acyl transferase. It has been reported that phenolic compounds can decrease LDL and increase HDL, which may hasten the removal of cholesterol from peripheral tissue to the liver for catabolism and excretion [48]. The combined effect of the polyphenolic compounds involved in the capping of iron oxide nanoparticles might have synergistically accounted for the observed decrease in total cholesterol, triglycerides, and LDL-cholesterol.

Table 3 shows the effect of iron oxide nanoparticles on liver enzymes. There was an alteration in enzyme activities as the levels of AST and ALT were significantly increased (*p* < 0.05) in group II (negative control) animals, but there was no significant change in the level of ALP after administration of 100 mg/kg body weight Triton X-100 compared to the group I (sham) animals. A similar finding was reported by Innih et al. [49], who found a significant increase in the serum levels of AST and ALT of rats after being administered with Triton. The increase in the activity of AST and ALT might indicate hepatocellular injury induced by Triton X-100 injection. This resulted in an increase in hepatic cholesterol biosynthesis and the accumulation of triglycerides in plasma. Induction of cellular linkages by high lipid accumulation in the hepatocytes of the liver parenchyma might be the cause of the observed increase. AST is less specific for liver disease, whereas ALT is known to increase in hepatic necrosis [49]. Treatment with iron oxide nanoparticles significantly decreased serum activities of AST and ALT in a dose-dependent manner, with the highest activity seen at 500 *µ*g/kg body weight, which may be suggestive of the iron oxide nanoparticle's effective dose. Iron oxide nanoparticles mitigated the effect of Triton X-100 as judged by the biochemical findings (reduction in the levels of serum AST, ALT, and ALP in a dose-dependent manner). The mechanism of action of iron oxide nanoparticles in ameliorating liver diseases could be attributed to their effectiveness in decreasing plasma lipids.

Table 4 shows the effect of iron oxide nanoparticles on serum antioxidant markers. The activities of SOD, CAT, and GPx were significantly decreased in the Triton X-100-treated group compared to the sham group. The antioxidant enzyme system is critical in terms of disease conditions. The system serves as a marker in monitoring oxidative stress during metabolic or disease conditions where they serve as a deterrent to the deleterious effect of free radicals. For instance, SOD catalyzes the dismutation of highly reactive superoxide anions (O_2_^−^) into O_2_ and hydrogen peroxide (H_2_O_2_) [50], which is less reactive, hence averting the deleterious effects of superoxide radical. The decrease in the activities of these antioxidant enzymes could be attributed to their exhaustive utilization in quenching the free radicals generated due to hyperlipidaemia as manifested by the high level of lipid peroxidation. Iron oxide nanoparticles increased the activities of CAT and SOD in Triton X-100-treated rats in a dose-dependent manner but had no significant effect on the activity of GPx. The increment in the activities of the antioxidant enzymes could be attributed to the high affinity binding of iron to both CAT and SOD. CAT is an important Fe-containing enzyme in blood. CAT is directly involved in the enzyme reaction that detoxifies H_2_O_2_, a damaging by-product of many metabolic processes in living organisms. CAT also helps to scavenge free radicals and increases antioxidative activity by decreasing their harmful effects on the host as well as supporting proper immune function [51].

Table 5 shows the effect of iron oxide nanoparticles on serum cardiovascular biomarkers. The activities of creatinine kinase, troponin I, and myoglobin were significantly increased (*p* < 0.05) in the Triton X-100-treated group (negative control) compared to the sham group. This result corroborated the findings of Suanarunsawat and colleaugues [52] who reported that high cholesterol markedly suppressed hepatic and cardiac functions as expressed by an elevation in the serum levels of AST, ALT, troponin, and creatinine kinase activities. Creatinine kinase, troponin I, and myoglobin are well-known diagnostic markers of myocardial damage. Administration of iron oxide nanoparticles was marked by a significant decrease (*p* < 0.05) in the levels of creatinine kinase, troponin, and myoglobin. The amelioration was done in a dose-dependent manner, with the highest activity seen in the group administered 500 *µ*g/kg body weight. The amelioration by iron oxide nanoparticles could be attributed to its effectiveness in inhibiting intracellular reactive oxygen species (ROS) as well as reducing the peroxidation of membrane lipids and lowering plasma lipids.

Figures 4(a)-4(f) show the effect of iron oxide nanoparticles on cardiac histopathology. The heart section of group 2 (negative control) showed marked fatty infiltration in cardiomyocytes and altered tissue architecture compared to group 1 (sham), which showed normal tissue architecture, no fatty changes, and normal morphology of myocardial cells. This result agrees with other studies showing that there is a relationship between hyperlipidaemia and pathological alteration of heart organs in rats [53]. Histopathological studies are the most precise evidence for the protective effect of drugs as protectants [54].

Although not completely restored to its normal state, administration of iron oxide nanoparticles reduced the fatty infiltration in cardiomyocytes seen in all treatment groups (100, 300, and 500 *µ*g/kg body weight FeO NPs) when compared to the negative group (group II) in a dose-dependent manner, with the highest dose of 500 *µ*g/kg body weight showing the most reduction, confirming its status as the most effective dose.

## 5. Conclusion

The results obtained from this study showed that iron oxide nanoparticles ameliorated the atherosclerotic effect of Triton X-100. The amelioration was dose-dependent, as seen above. The maximal effect was achieved at 500 *µ*g/kg body weight concentration of iron oxide nanoparticles as observed in all parameters that were assayed for, confirming its status as the most effective and suitable dose for treatment. Although not as effective as the standard drug atorvastatin when compared, iron oxide nanoparticles synthesized using the leaf extract of *Spinacia oleracea* can serve to reduce the atherosclerotic effect of Triton X-100. Our discovered antiatherosclerotic activity of FeO NPs may have a clinical implication because the NPs are easier to prepare under the condition of avoiding environmental contamination. We expect that application of FeONPs to treat cardiovascular diseases would significantly reduce the costs associated with currently used drugs that are made from either natural sources or chemical synthesis.

## Figures and Tables

**Table 1:** DPPH radical scavenging activity of iron oxide nanoparticles (% inhibition).

Concentration (*µ*g/ml)	FeO NPs	Ascorbic acid
20	35.08 ± 0.92^a^	46.04 ± 0.70
40	42.16 ± 0.91^a^	58.23 ± 0.79
60	53.81 ± 1.64^a^	66.05 ± 0.78
80	60.22 ± 0.52^a^	75.36 ± 0.52
100	76.33. ±0.57^a^	87.81 ± 0.30

Values are Mean ± SEM (*n* = 5). a = significantly (*p* < 0.05) lower than ascorbic acid at the same concentration.

**Table 2:** Effect of iron oxide nanoparticles on lipid profile parameters in mg/dL.

Group	TC	TG	HDL	LDL	AI
Normal	110.36 ± 1.48^bf^	87.35 ± 0.87^bf^	69.06 ± 0.95	23.83 ± 1.11^bf^	0.59 ± 0.03^bf^
Negative	245.26 ± 1.73^ac^	220.75 ± 1.34^ac^	20.03 ± 0.39^d^	181.08 ± 1.31^ac^	11.24 ± 0.02^ac^
Standard (atorvastatin	114.26 ± 1.78^bjf^	90.85 ± 1.48^bjf^	71.27 ± 1.43	25.22 ± 1.16^bjf^	0.60 ± 0.04^bf^
100 *µ*g/kg bw FeO NPs	191.02 ± 1.69^abc^	170.51 ± 0.89^abc^	50.05 ± 0.06d^ej^	106.85 ± 1.50^abc^	2.82 ± 0.05^abc^
300 *µ*g/kg bw FeO NPs	156.07 ± 0.32^abc^	134.02 ± 1.09^abc^	59.22 ± 0.35^dej^	70.05 ± 0.65^abc^	1.64 ± 0.04^abc^
500 *µ*g/kg bw Fe ONPs	121.91 ± 1.24^abcf^	98.27 ± 1.37^abcf^	68.70 ± 0.26^de^	33.56 ± 1.19^abcf^	0.77 ± 0.05^abc^

Values are Mean ± SEM (*n* = 5). a = significantly (*p* < 0.05) higher than sham. b = significantly (*p* < 0.05) lower than negative control. c = significantly (*p* < 0.05) higher than standard control. d = significantly (*p* < 0.05) lower than normal control. *e* = significantly (*p* < 0.05) lower than standard control. f = significantly (*p* < 0.05) lower than other treatment groups.

**Table 3:** Effect of iron oxide nanoparticles on liver biomarkers in U/L

Group	ALT	AST	ALP
Normal	28.65 ± 0.21^bd^	33.91 ± 0.88^bd^	89.45 ± 1.16
Negative	74.59 ± 0.91^ac^	67.93 ± 0.96^ac^	98.13 ± 1.12
Standard (atorvastatin)	28.95 ± 0.98^bd^	35.24 ± 0.18^bd^	88.26 ± 1.16
100 *µ*g/kg bw Fe ONPs	50.90 ± 0.65^abc^	60.43 ± 0.87^abc^	95.54 ± 0.72
300 *µ*g/kg bw Fe ONPs	46.11 ± 0.89^abc^	53.07 ± 0.65^abc^	92.94 ± 0.38
500 *µ*g/kg bw Fe ONPs	39.30 ± 0.72^abcd^	44.25 ± 1.06^abcd^	90.69 ± 0.70

Values are Mean ± SEM (*n* = 5). a = significantly (*p* < 0.05) higher than sham. b = significantly (*p* < 0.05) lower than negative control. c = significantly (*p* < 0.05) higher than standard control. d = significantly (*p* < 0.05) lower than other treatment groups.

**Table 4:** Effect of iron oxide nanoparticles on serum antioxidant markers.

Group	SOD units per millilitre (U/ml)	CAT micromole per hydrogen peroxide per minute (µmol/H_2_O_2_/min)	GPx units per millilitre (U/ml)
Normal	2.99 ± 0.05^b^	57.58 ± 0.84^b^	36.03 ± 2.97^b^
Negative	1.31 ± 0.05^adc^	30.72 ± 0.74^adc^	19.43 ± 0.60^ac^
Standard (atorvastatin)	2.93 ± 0.05^b^	56.02 ± 0.49^b^	39.44 ± 0.49^b^
100 *µ*g/kg bw Fe ONPs	1.99 ± 0.03^ab^	44.35 ± 0.25^ab^	20.08 ± 0.33^ac^
300 *µ*g/kg bw Fe ONPs	2.23 ± 0.04^ab^	49.81 ± 0.39^ab^	20.99 ± 0.37^ac^
500 *µ*g/kg bw Fe ONPs	2.89 ± 0.04^b^	55.69 ± 0.46^b^	22.02 ± 0.66^ac^

Values are mean ± SEM (*n* = 5). a = significantly (*p* < 0.05) lower than sham. b = significantly (*p* < 0.05) higher than negative control. c = significantly (*p* < 0.05) lower than standard control. d = significantly (*p* < 0.05) lower than other treatment groups.

**Table 5:** Effect of iron oxide nanoparticles on serum cardiovascular markers in ng/ml.

Group	CK-MB	CTnI	Myoglobin
Normal	3.67 ± 0.09^bd^	0.03 ± 0.01^bd^	56.46 ± 2.31^bd^
Negative	12.70 ± 0.29^ac^	0.87 ± 0.10^ac^	93.64 ± 1.54^ac^
Standard (atorvastatin)	3.89 ± 0.08^b^	0.05 ± 0.01^b^	59.49 ± 1.94^b^
100 *µ*g/kg bw FeO NPs	8.87 ± 0.09^abc^	0.23 ± 0.06^abc^	86.34 ± 0.60^ac^
300 *µ*g/kg bw FeO NPs	6.95 ± 0.04^abc^	0.16 ± 0.08^abc^	70.40 ± 0.55^abc^
500 *µ*g/kg bw FeO NPs	4.86 ± 0.27^bcd^	0.09 ± 0.02^bd^	61.96 ± 1.24^bd^

Values are Mean ± SEM (*n* = 5). a = significantly (*p* < 0.05) higher than normal control. b = significantly (*p* < 0.05) lower than negative control. c = significantly (*p* < 0.05) higher than standard control. d = significantly (*p* < 0.05) lower than other treatment groups.

## Data Availability

The dataset that supported the conclusion of this article is included within the article. However, additional data, if required, are available upon request.
